# ECG signal classification based on deep CNN and BiLSTM

**DOI:** 10.1186/s12911-021-01736-y

**Published:** 2021-12-28

**Authors:** Jinyong Cheng, Qingxu Zou, Yunxiang Zhao

**Affiliations:** School of Computer Science and Technology, Qilu University of Technology (Shandong Academy of Sciences), Jinan, China

**Keywords:** Atrial fibrillation, DCNN, BiLSTM, TMSE

## Abstract

**Background:**

Currently, cardiovascular disease has become a major disease endangering human health, and the number of such patients is growing. Electrocardiogram (ECG) is an important basis for {medical doctors to diagnose the cardiovascular disease, which can truly reflect the health of the heart. In this context, the contradiction between the lack of medical resources and the surge in the number of patients has become increasingly prominent. The use of computer-aided diagnosis of cardiovascular disease has become particularly important, so the study of ECG automatic classification method has a strong practical significance.

**Methods:**

This article proposes a new method for automatic identification and classification of ECG.We have developed a dense heart rhythm network that combines a 24-layer Deep Convolutional Neural Network (DCNN) and Bidirectional Long Short-Term Memory (BiLSTM) to deeply mine the hierarchical and time-sensitive features of ECG data. Three different sizes of convolution kernels (32, 64 and 128) are used to mine the detailed features of the ECG signal, and the original ECG is filtered using a combination of wavelet transform and median filtering to eliminate the influence of noise on the signal. A new loss function is proposed to control the fluctuation of loss during the training process, and convergence mapping of the tan function in the range of 0–1 is employed to better reflect the model training loss and correct the optimization direction in time.

**Results:**

We applied the dataset provided by the 2017 PhysioNet/CINC challenge for verification. The experiment adopted ten-fold cross validation,and obtained an accuracy rate of 89.3$\%$ and an F1 score of 0.891.

**Conclusions:**

This article proposes its own method in the aspects of ECG data preprocessing, feature extraction and loss function design. Compared with the existing methods, this method improves the accuracy of automatic ECG classification and is helpful for clinical diagnosis and self-monitoring of atrial fibrillation.

## Background

Atrial Fibrillation (AF) is very common in patients with organic heart disease, and can lead to a variety of complications [1]. A large number of patients with paroxysmal atrial fibrillation generally have no specific symptoms in daily life, which is easy to delay diagnosis and lead to aggravation [2]. AF can increase the risk of heart failure and stroke [3]. In addition, the duration of paroxysmal atrial fibrillation is short and does not continue to show abnormalities on the ECG. Routine ECG examination is only a few minutes. For patients with paroxysmal atrial fibrillation, it often leads to missed diagnosis and misdiagnosis [4]. Dynamic ECG machine is used to detect ECG changes for a long time, and capture abnormal ECG when AF occurs to reduce the rate of missed diagnosis [5]. However, the long-term detection and lengthy ECG data greatly reduce the diagnostic efficiency of the medical doctor and increase the workload of the medical doctor. Therefore, the automatic ECG analysis and classification algorithm has important clinical value.

ECG devices are often more sensitive when collecting ECG signals and contain a variety of noises [6]. In dynamic electrocardiogram, signal noise often occurs at a certain moment. Different types of noise include contact noise, Electro-Magnetic Gun (EMG)noise, baseline drift, power line interference and motion artifacts, etc. [7, 8]. The preprocessing of the ECG signal must use the denoising method of the ECG signal, and different software algorithms can be used to process and eliminate these noise sources. Because the ECG signal is relatively weak, these noises can interfere with useful information [9, 10]. Therefore, we need to preprocess the original ECG before extracting features to filter out noise. Many scholars at home and abroad have done a lot of work on denoising ECG signals. Sharma uses a dualband filter bank combined with a product filter and a lowpass filter to perform noise reduction on the ECG signal [11]. Peng Xiong uses a combination of wavelet scale adaptive threshold method and Denoising Automatic Encoder (DAE) to eliminate the influence of noise in the ECG signal, and cooperate with the Deep Neural Network (DNN) to extract features, the results show that the method shows a significant improvement in signal-to-noise ratio and root mean square error [12]. Alickovic E uses Multi-Scale Principal Component Analysis (MSPCA) to filter out the noise in ECG signals in data preprocessing, but MSPCA is not as common as other research fields in the field of ECG classification [13]. Phuong uses a combination of integrated empirical mode decomposition and genetic algorithm threshold technology to perform noise reduction on ECG signals. However, the genetic algorithm may obtain a local optimal solution during the optimization process [14].

The ECG signal automatic classification system has high practical value and is one of the hotspots of current research. With the continuous development of deep learning, deep learning has performed well in mining complex structures in High-Dimensional data [15].Martinez first applied deep learning to the classification and recognition of physiological signals, and achieved 70–75% accuracy [16]. Zidelmal et al. detected and segmented QRS complexes on the MIT-BIH arrhythmia database. The author first uses a set of features to represent each beat, such as frequency information, RR interval, QRS shape, and QRS detail coefficient. The second step uses Support Vector Machine (SVM) to classify feature vectors.Experimental results show that the average accuracy of this method is 97.2$\%$ [17]. However, in the actual application process, it is found that the use of SVM to classify high-dimensional data requires higher computer hardware, and the reduction of data dimension classification will reduce the classification accuracy due to missing data dimensions. Goodfellow, Sebastian D proposed to use 13layer convolutional neural network (CNN) to extract ECG signal features, and use softmax classifier for classification. Manuscript File Click here to view linked References. The f1 score was 0.84 on the ECG data set provided by 2017 PhysioNet/CINC challenge [18].This method needs to segment the data first, which undoubtedly increases the uncertainty in the segmentation process and the test of the reliability of the segmentation results. B Pyakillya uses a convolutional neural network with a 1D convolutional layer on the 2017 PhysioNet/CINC challenge data set to identify and classify ECG signals. The accuracy of the learning framework in verifying the best results of data is 85.5$\%$ [19].In the in-depth analysis of the ECG signal, we found that this signal has a very strong timing characteristic, which is congenital and an indispensable diagnostic factor. However, in the method of [20], not found use the timing information of ECG signals.

Hannun have developed a deep neural network for classifying 12 types of ECG signals in single-lead ECG signals and comparing their classification performance with the results of cardiologists. The accuracy of using DNN is as high as 83.7$\%$, exceeding 78$\%$ of human cardiologists [21]. It can be seen from the above literature that the use of deep learning framework for automatic recognition and classification of ECG signals continues to advance, but the recognition accuracy of the automatic ECG classification system is not high, and there is room for improvement.SmisekIt uses the local signal information of the ECG signal and the entire ECG signal record to extract the characteristic information of the ECG signal, and uses a combination of support vector machines, decision trees and threshold-based rules to identify and classify the ECG [22].Rubin uses the signal quality index to evaluate noise during the data pre-processing stage, and uses two convolutional neural networks to extract different lengths of ECG signal feature information, and then sends the feature information to the classifier for classification [23].Xiong proposed to use 21-layer 1D convolutional recurrent neural network to extract features of ECG, expand the convolution filter to increase local perception, and use residual connection, normalization and other methods to improve the efficiency of the algorithm [24].Teijeiro uses consensus-cantiveframework to extract a set of ECG signal features to describe the morphological characteristics of the entire ECG signal. It marks the ECG record twice and cooperates with the recurrent neural network to increase the accuracy of recognition classification [25].Rizwan uses ECG waveforms to extract ECG signal features, uses feature selection methods for feature dimensionality reduction, and finally uses ECG to classify ECG signals [26].

In this study, we focus on the characteristics that the ECG signal is weak and easy to be disturbed, and the problem that the extracted feature level is insufficient to cause low recognition classification accuracy, mainly in the following aspects:

- We used the ECG data set provided by the 2017 PhysioNet / CinC Challenge, and screened 8528 pieces of data. Based on the sampling points of the data set, we filtered out some data with less data features, and used 7561 pieces of data for training tests The data is cleaned, and the wavelet transform (WT) and median filter (MT) are used to perform the filtering process, which effectively retains the signal characteristic value and has better noise removal effect.
- In view of the more verbose characteristics of ECG signals in time series, DCNN can be used to mine deep-level ECG data features, and BiLSTM can also take into account the time-sensitive feature information of ECG data In this paper, a DCNN and BiLSTM network model are used for feature extraction, which can extract more complete ECG signal features and provide more complete information for the classifier.
- We improved the loss function and proposed a new loss function to optimize the ECG signal classification model. Through our proposed loss function, we can better reflect the loss of model training to timely modify the optimization direction and effectively improve model recognition classification accuracy.

## Related research

The topic of deep learning is the study of knowledge extraction, prediction, and intelligent decision-making, or the use of a set of main sentences, which is the so-called training data, to identify complex patterns. In recent years, in order to improve the accuracy of different learning tasks, people have proposed several deep learning models, including multi-layer perceptron, CNN, LSTM and Deep Belief Network (DBN).CNN is generally composed of multiple convolutional layers and pooling layers. The convolutional layer continuously updates the weight matrix through training. In the optimization process, the same feature plane shares the weight to reduce the amount of calculation and reduce the fit. The pooling layer is used to simplify the model complexity, and sub-sampling is used to filter out some parameters to improve the calculation efficiency of the model [17, 27]. Finally, a reasonable weight matrix is obtained to extract the ECG data features. The structure of the convolutional neural network is shown in Table 1.

Input:

1 $$\begin{aligned} V=conv(W,X, ``valid'')+b \end{aligned}$$

Output:

2 $$\begin{aligned} Y=\varphi (V) \end{aligned}$$

The above input and output formulas are for a convolutional layer. Each convolutional layer has a different weight matrix W, and W, X, and Y are in the form of a matrix. For the last fully connected layer, set it to the Lth layer, the output is $y^L$ in vector form, and the expected output is d, then there is the total error formula:

3 $$\begin{aligned} E=\frac{1}{2}\Vert {d-y^L}\Vert ^2_2 \end{aligned}$$

where conv is the convolution operation function, the second parameter valid indicates the type of convolution operation, the Formula (1) is the valid type, X represents the input matrix, W represents the weight matrix of the convolution kernel, b represents the offset term, $\varphi (x)$ is the activation function, d and y in the total error are the vector of the expected output and the network output, $\Vert {x}\Vert _2$ is the 2-norm of the vector x.

LSTM is widely used in the field of natural language processing, and has a good experimental effect on the classification of sequence data. ECG signal classification is to classify a given ECG signal. Since the ECG signal has obvious timing characteristics, it can be regarded as a classification problem of sequence data. BiLSTM consists of two layers of LSTM networks, both of which have input sequences, but the direction of information transmission is opposite. BiLSTM includes a forward LSTM and a reverse LSTM. In the Formula (7), hat represents the forward LSTM output, and in the Formula (8) hbt represents the reverse LSTM output. In the Formula (9), the two hidden state vectors extracted from the forward and reverse directions are connected to integrate the characteristics of the front and back ECG signals.So this experiment chooses Bidirectional long-short-term memory recurrent network (BiLSTM) as the training model.

4 $$\begin{aligned} {h_{at}}= & {} f({U_a}{h_{a(t - 1)}} + {W_a}{x_t}]) \end{aligned}$$

5 $$\begin{aligned} {h_{bt}}= & {} f({U_b}{h_{b(t + 1)}} + {W_b}{x_t}]) \end{aligned}$$

6 $$\begin{aligned} {h_t}= & {} {h_{at}} \oplus {h_{bt}} \end{aligned}$$

## Model

### Our model

Aiming at the characteristics of weak ECG data signal and more verbose in time series, we designed a neural network combining deep convolution and BiLSTM to extract features. The convolutional neural network has the characteristics of local receptive field and weight sharing. Each neuron does not need to feel all the signals, but only needs to feel the local characteristics. Then at a higher level, the different local neurons obtained by these feelings can be synthesized to obtain global information. The sharing of parameters between different neurons can reduce the parameters that need to be solved, and using multi-layer convolution will get a variety of feature maps. Weight sharing is actually a convolution operation on the ECG signal with the same convolution kernel, so that all neurons in the first hidden layer can detect features in different positions of the signal. Convolutional neural networks can reduce the number of connections and deepen the network structure to better mine data features.

Compared with the convolutional neural network model used for image classification, compared with the extraction of image data, this paper uses a larger convolution kernel to expand the perception field of view of the convolution kernel. The network depth is deepened, and 24 convolutional layers are designed. Each eight-layer convolutional neural network adopts different convolution kernel sizes to better extract data feature values. The number of convolution kernels is set to 32, 64, and 128 for every eight layers. For the ageing characteristics of ECG data, a larger convolution kernel is adopted than for ordinary image data, and the size of each layer is set to 16. The size and number of different convolution kernels are used, and the Relu activation function is added after each convolution layer to alleviate the gradient disappearance problem and can converge faster. After every two convolutional layers, a pooling layer is added. The pooling layer is mainly used for feature dimensionality reduction, compressing the number of data and parameters, reducing overfitting, and improving the fault tolerance of the model. The pooling layer is a vector used for scalar transformation of each local area of the data like convolution, ensuring the efficiency of the algorithm. In order to prevent the model from overfitting, and add dropout after each convolutional layer, it can randomly set some activation values to 0, forcing the network to explore more ways to classify data, rather than over-relying on some functions. And add batch normalization (BN) after each convolutional layer. During model training, batch normalization uses the mean and standard deviation of small batches to continuously adjust the intermediate output of the neural network. Such an operation can make the intermediate output value of the entire neural network more stable. Prevent deep neural networks from encountering slow convergence, or gradient explosions and other problems that cannot be trained.

The LSTM adds a self-feedback connection. The current state of the neuron is determined by the input and the last state of the neuron. Units with influence on subsequent states can be remembered, which is very suitable for ECG data with longer timeliness. The BiLSTM used in this paper is a combination of forward LSTM and backward LSTM. BiLSTM can better capture the bidirectional data dependence. In this paper, a DCNN combined with BiLSTM model is used to extract data features, which can better mine data features and obtain better classification accuracy.The model network structure is shown in Table 2.

**Table 1:** Structure diagram of convolutional neural network

Input layer		
conv1D-16 conv1D-16 MaxPoling1D		
conv1D-64 conv1D-64 MaxPoling1D		
conv1D-128 conv1D-128 MaxPoling1D		
conv1D-256 conv1D-256 MaxPoling1D		
GlobalAveragePooling1D		
Dropout		
Dropout		
Output layer

**Table 2:** Network structure

Input layer		
Conv1D-32+ Relu	X2	X4
Batch Normalization		
Dropout		
Conv1D-64+ Relu	X2	X4
Batch normalization		
Dropout		
Conv1D-128+ Relu	X2	X4
Batch Normalization		
Dropout		
BiLSTM-64 BiLSTM-128 Dense(128)+Relu Dense(64)+Relu Dense(32)+Relu Dense(4)+Relu		X1
Output Layer		

### Loss function

The loss function is an indicator to measure the performance of the prediction model to predict the expected results. Based on the mean square error loss function (MSE), this paper proposes a TMSE loss function for the calculation of ECG data loss, as shown in Formula (10). For the mean square error function, if the error value has an outlier, the loss model will be given a higher weight to the outlier. The value of the error calculated by the loss function will increase a lot. Because ECG data will have uncontrollable noise interference during the collection, singular points and outliers will often appear. For the error characteristics extracted during ECG data training, we increase the tan function to control the error when taking the mean square error too large or too small floats, through the mapping of the tan function, can effectively suppress the influence of outliers on the entire model, get a more stable loss calculation, and adjust the direction and magnitude of gradient descent.

**Fig. 1:**
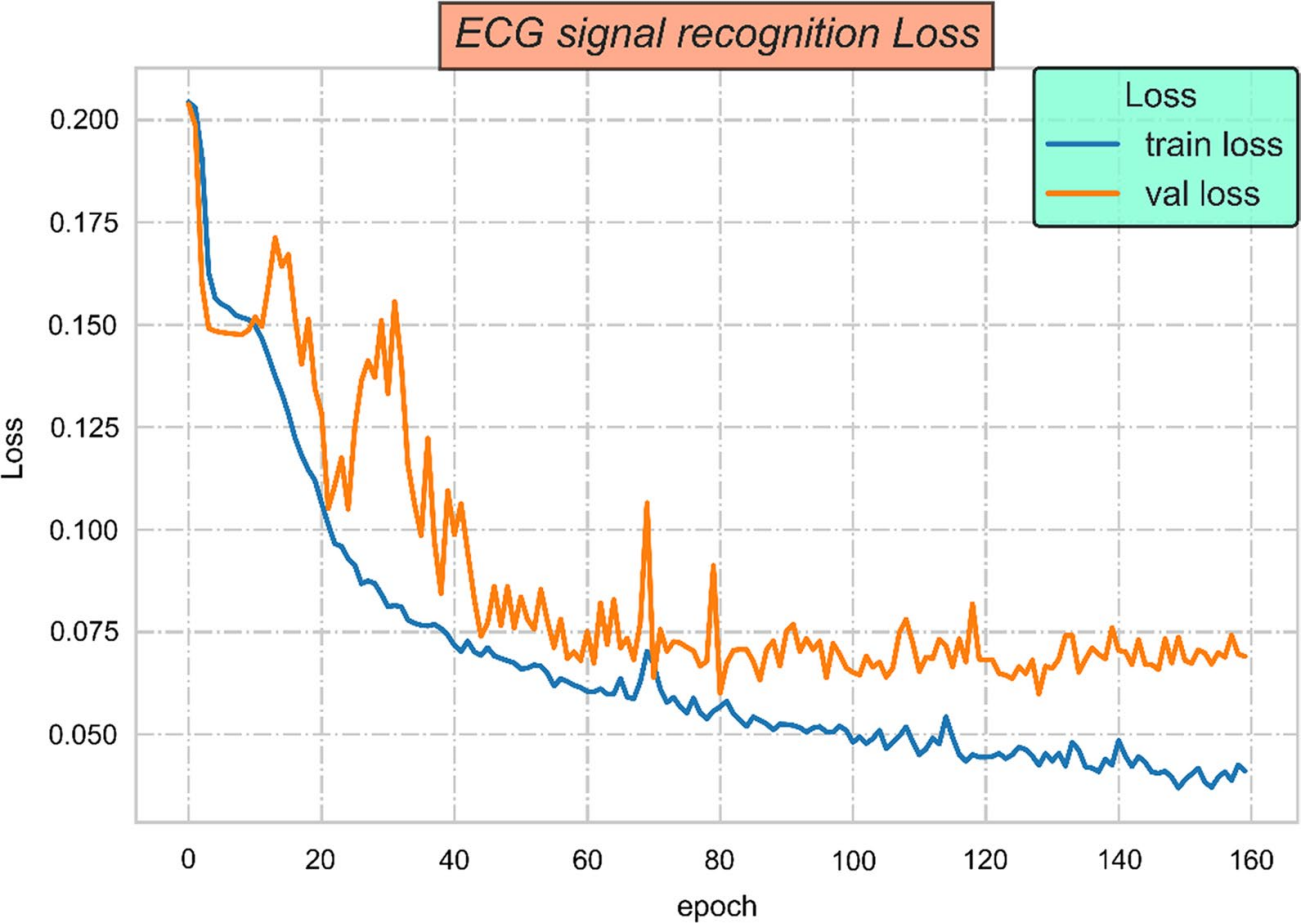
Loss function graph

The loss function we proposed will adjust the model to minimize outlier data points and have better and more robustness to outlier points. Through experimental comparison, it is proved that the loss function we proposed has a significant effect on improving the accuracy of ECG classification. Figure 1 is a training and test loss function diagram. After the loss function we proposed to control, there are outliers and singular points in the ECG data. Through the mapping and control of the loss function we proposed, the loss function curve gradually stabilizes and the error float also tends to be stable.

7 $$\begin{aligned} MSTE=\frac{1}{N}\sum \limits _{i=1}^Ntan((y_i-\widehat{y_i})^2) \end{aligned}$$

## Experiment and results

### DataSet and experimental settings

In this paper, we used the ECG dataset provided by the 2017 PhysioNet/CINC Challenge [28]. Table 3 shows the data distribution in this data set. The data set contains a total of 8528 single-lead data. The shortest electrocardiogram record is 9s and the longest 61s. It is mainly divided into four types of ECG: AF, other heart diseases, noise and normal sinus rhythm. Each ECG record contains two texts: the .mat file contains the characteristic information of the ECG data, and the .hea file contains the interpretation information of the ECG record. We screened based on the average length of the data, screened out too short ECG signals, and finally used 7561 pieces of data for training and testing. We take 90% of the ECG data for training, and the remaining data is used as a test set to test the model.We use Keras to build our network architecture, and provide measurement functions for evaluation indicators, and the experiment sets tenfold cross-validation. The experimental hardware configuration is Intel(R) Xeon(R) Gold 5118 CPU @ 2.30GHz and two RTX 2080Ti GPUs.

**Table 3:** ECG data description

Type	Time length (s)					
	#recording	Mean	SD	Max	Median	Min
Normal	5154	31.9	10.0	61.0	30	9.0
AF	771	31.6	12.5	60	30	10.0
Other rhythm	2557	34.1	11.8	60.9	30	9.1
Noisy	46	27.1	9.0	60	30	10.2
Total	8528	32.5	10.9	30	30	9.0

### Data preprocessing

The data set contains 8528 single-lead ECG records with data dimensions ranging from 2700 to 18,000. Because the data dimension gap is too large, in order to better extract data features for training, we set the minimum data dimension data selection was conducted for 9000, and 7561 data were used as the data set for our experiment.

**Fig. 2:**
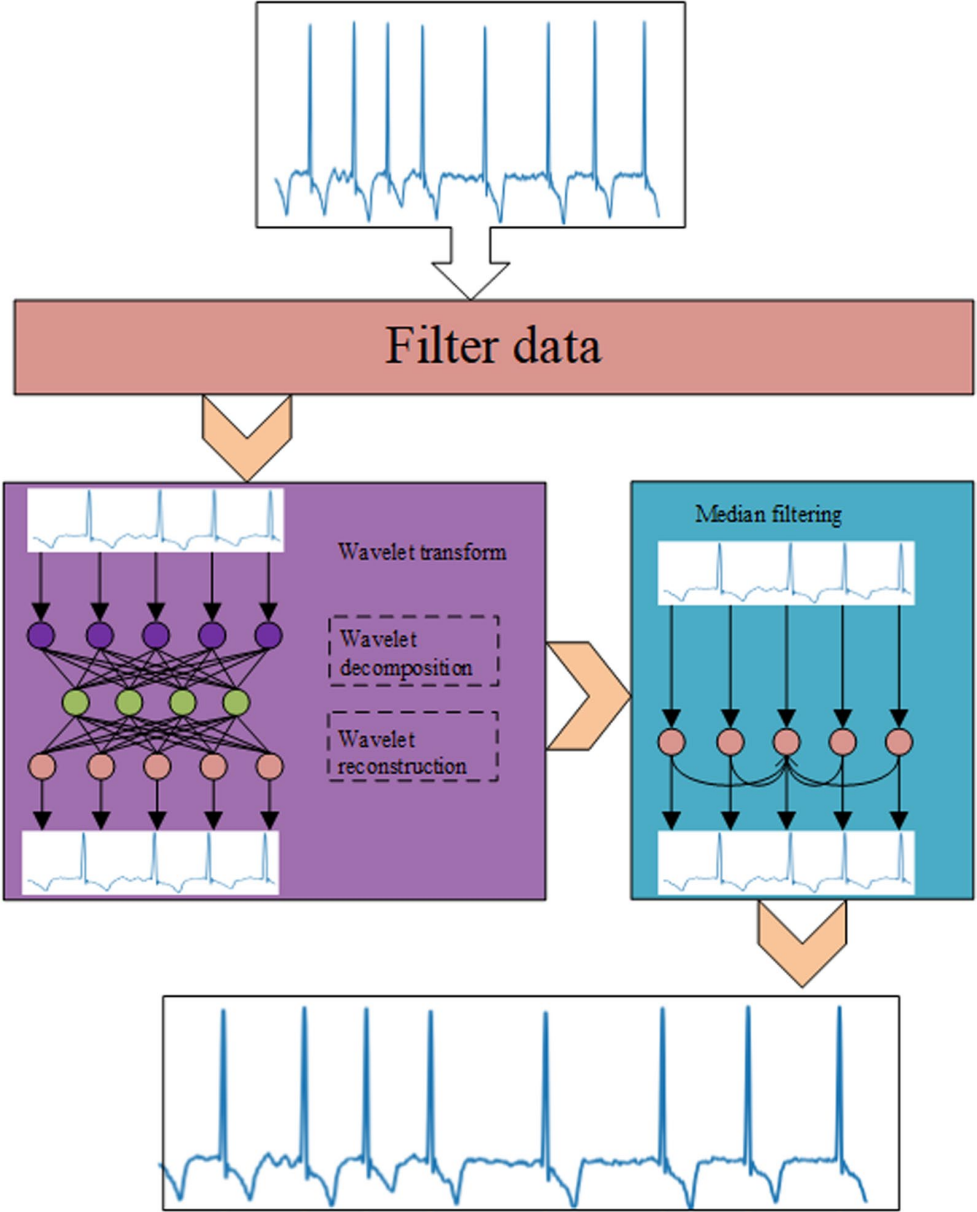
Filtering flowchart

Noise interference has a comprehensive effect on all biomedical signals. Therefore, strict preprocessing is required in the data preprocessing stage. First of all, we screened the ECG signal, and combined the wavelet transform filter and the median filter on the ECG to reduce the interference of the ECG identification and classification as much as possible. The filtering process is shown in Fig. 2.

First of all, we filter the ECG signal by wavelet transform. Through the expansion and translation of the basis function, it can have good localization properties in the time and frequency domain at the same time, so as to filter out the interference waveform in the ECG data. The wavelet transform function, such as Formula (11). Where, we use the scale $\alpha$ to transform the wavelet function, and use the shift amount $\iota$ to translate the wavelet function. The ECG signal is decomposed. In this study, the decomposition parameter is set to 9, and the original signal is decomposed into wavelet components to the selected layer. After noise filtering, the signal is reconstructed by wavelet to obtain ECG signals of different scales.

8 $$\begin{aligned} W,T(\alpha ,\iota )=\frac{1}{\sqrt{\alpha }}\int _{ - \infty }^{ + \infty }f(t)*\psi \left( \frac{t-\iota }{\alpha }\right) dt \end{aligned}$$

**Fig. 3:**
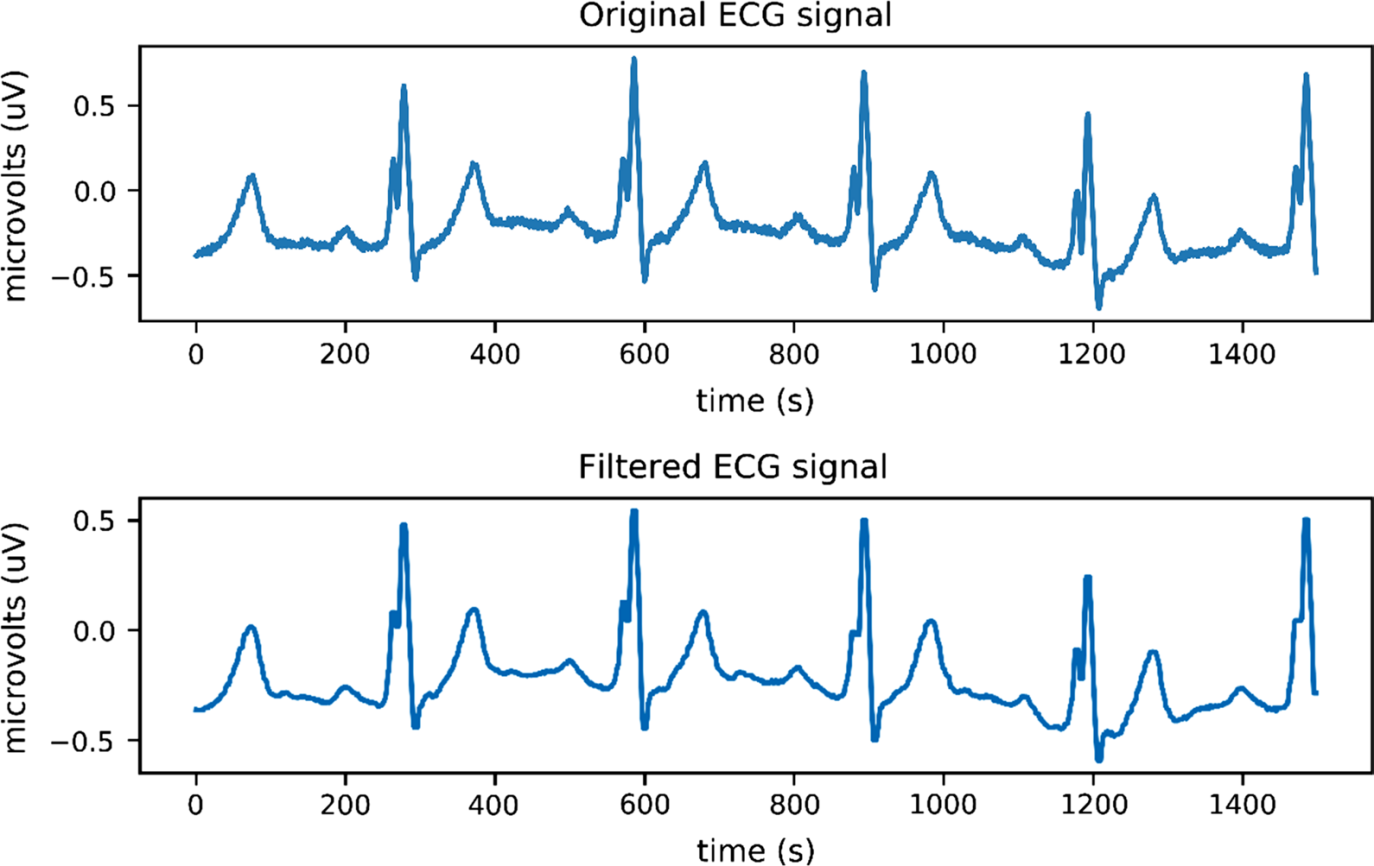
Filter comparison chart

In addition, we use median filtering to eliminate baseline drift noise. Median filtering is a non-linear digital filtering technique that has the characteristics of noise suppression and edge protection. To eliminate noise using median filtering, first remove the larger value in the ECG signal to obtain a trend term signal containing only the baseline, and then superimpose it with the original signal to eliminate the baseline drift interference in the original ECG signal. The neighborhood size is set to 9, that is, each point and four points on the left and right become a neighborhood. Select the median in the neighborhood of each position to replace the number in this position to eliminate the noise in the signal. Figure 3 is a comparison chart before and after filtering. The ECG signal after filtering is more stable.

### Evaluation standard

**Fig. 4:**
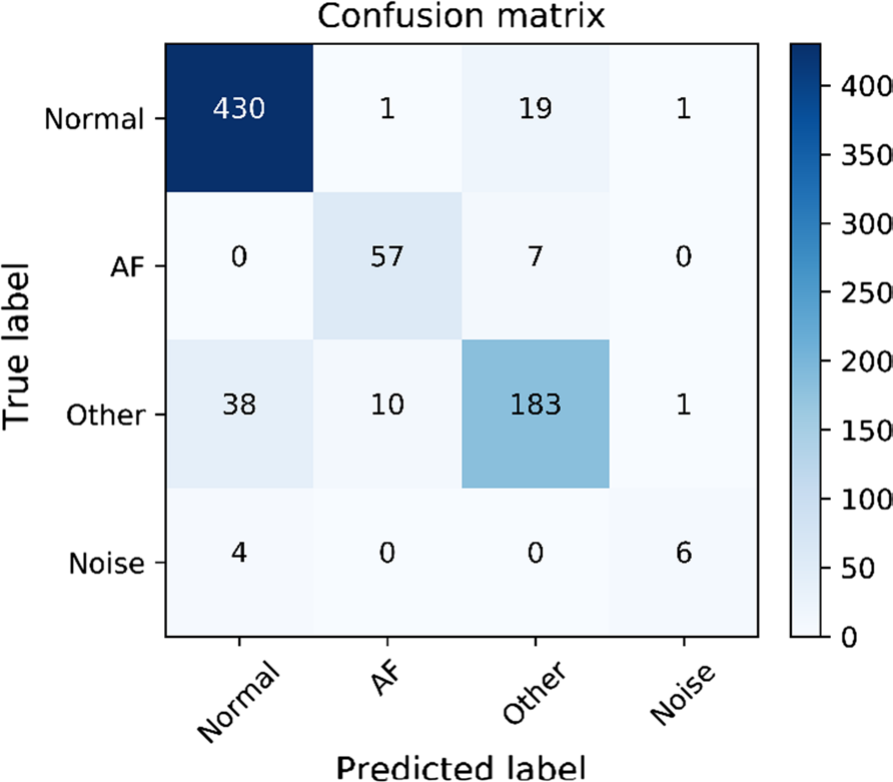
Confusion matrix

In this experiment, we adopt three-level evaluation indicators to evaluate the classification effect of the model. The first-level evaluation uses a confusion matrix (also called error matrix, Confusion Matrix) to display the classification effect [25]. The confusion matrix is mainly calculated based on four parameters: the true value is positive, and the ECG classification model considers the number of positives (True Positive = TP). The true value is positive, and the ECG classification model is considered to be negative (False Negative = FN). The true value is negative, and the ECG classification model considers it to be a positive number (False Positive = FP). The true value is negative, and the ECG classification model considers the number of negative (True Negative = TN). The four indicators are presented together in a table, showing a confusion matrix, as shown in Fig. 4. The confusion matrix counts the number. Sometimes it is difficult to measure the pros and cons of the model by simply counting the numbers. Therefore, the confusion matrix extends the secondary index accuracy (Accuracy) in the basic statistical results [26]. Through the secondary index, the result of the quantity in the confusion matrix can be converted into a ratio between 0–1. As in Formula (9), the accuracy rate in the second-level evaluation index is adopted to evaluate the entire model, which is convenient for standardized measurement. The three-level evaluation index F1-score is used to evaluate the classification performance, and the calculation formula is as shown in Formula (11). Among them, P stands for Precision, R stands for Recall in the secondary index. Among them, the Recall calculation formula is as shown in Formula (10), and the value range of F1-score is from 0 to 1, 1 represents the best output of the model, and 0 represents the worst output result of the model.

9 $$\begin{aligned} Accurary= & {} \frac{TP+TN}{TP+TN+FP+FN} \end{aligned}$$

10 $$\begin{aligned} Recall= & {} \frac{TP}{TP+FN} \end{aligned}$$

11 $$\begin{aligned} F1-score= & {} \frac{2PR}{P+R} \end{aligned}$$

### Method comparison

We conducted five sets of experiments to verify the effectiveness of the method:

First of all, the preprocessing stage of our experiment uses wavelet transform alone, and the neural network combined with deep convolution and BiLSTM in the network classification stage. At the same time, in conjunction with our proposed WT loss function, we call this experiment WT-TMSE; In the MT-TMSE experiment, the wavelet transform used in preprocessing was changed to median filter transform on the basis of WT-TMSE; The preprocessing stage of the WT-MT-CEEF experiment uses a filtering method combining wavelet transform and median filtering, with our proposed network structure and cross entropy loss function (CEEF); WT-MT-MSE changes the loss function to tan mean square error loss function based on the previous experiment; The WT-MT-TMSE experiment uses a filtering method combining wavelet transform and median filtering in the preprocessing stage, and uses the network model and TMSE loss function proposed in this paper. It is worth noting that the five experiments we designed all use the data set mentioned above and the same experimental environment configuration. Experiments show that the preprocessing of this paper uses a combination of WT and MT, feature extraction uses the network structure of the DCNN and BiLSTM designed by us, the loss function we propose can effectively improve the accuracy of ECG data classification.

**Fig. 5:**
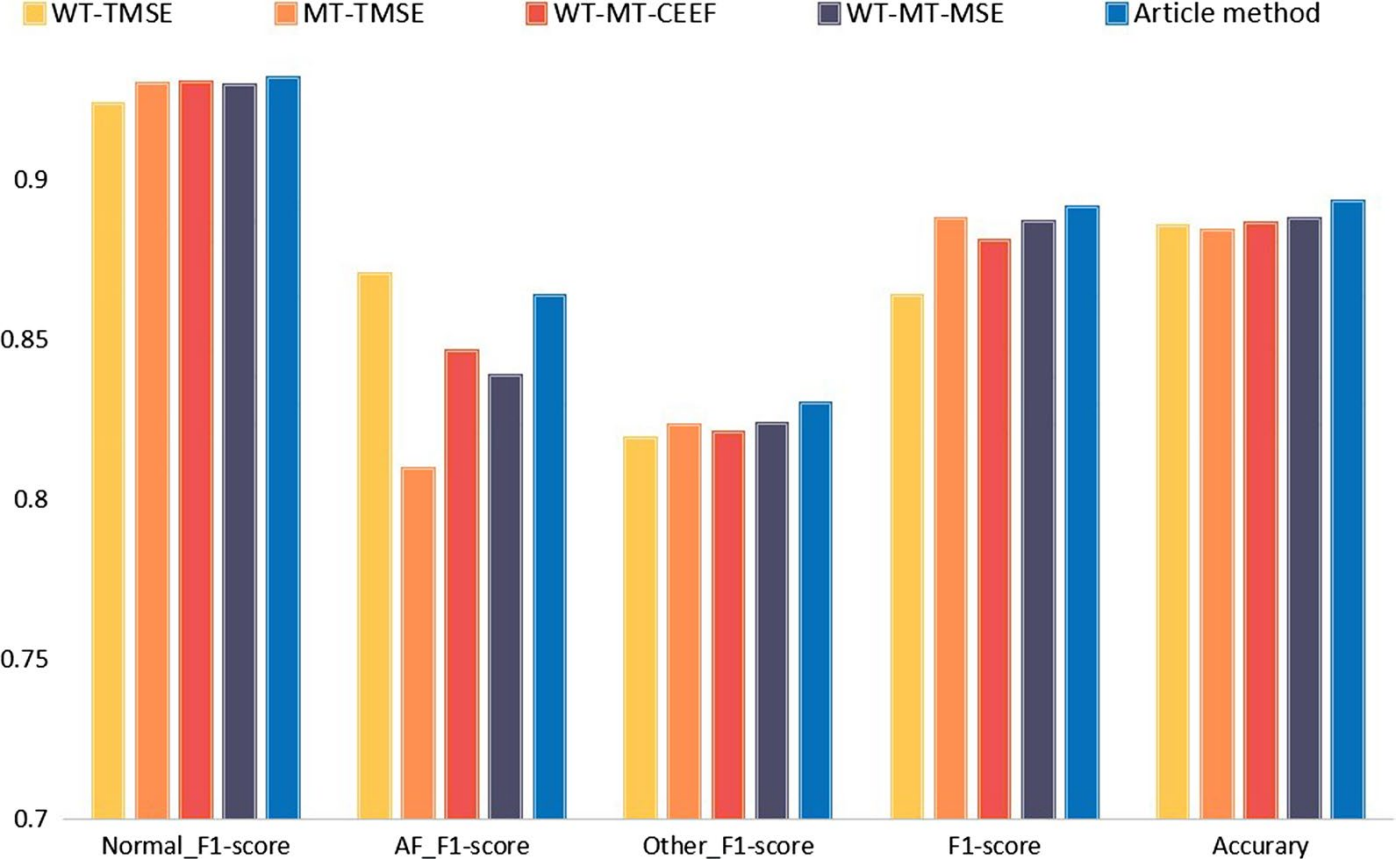
Experimental comparison chart

WT-TMSE uses WT alone to preprocess the ECG signal. In WT-TMSE, the F1-score of the AF ECG is slightly higher than the method in this chapter. The analysis in this article is to add the median filter to the part of the atrial fibrillation ECG. The QRS widening and deformation in the data has a certain inhibitory effect, but the accuracy of the F1-score of other types of ECG and the overall classification of the model is not as good as the method in this chapter; MT-TMSE uses median filtering alone to preprocess the ECG signal. The F1-score of the ECG data of the AF category is significantly lower than that of WT-TMSE, and the F1-score of the other two categories are both improved. It shows that the pros and cons coexist when the median filter is used to preprocess the ECG signal, which verifies the necessity of the combined filtering method proposed in this chapter; Compared with WT-MT-CEEF and WT-MT-MSE, WT-MT-TMSE has improved F1-score for Normal ECG, AF ECG and Other ECG. According to the loss characteristics of the ECG signal training process, it is shown that the improved TMSE loss function can better suppress the outliers in the ECG signal, so that the model can extract the characteristics of the ECG signal more accurately. This experiment verifies the effectiveness of the TMSE loss function.The experimental comparison histogram is shown in Fig. 5, and the experimental data comparison is shown in Table 4.

**Table 4:** Experimental classification results

Method	F1-score				Accurary
	Normal	AF	Other	Overall	
WT-TMSE	0.924	0.870	0.819	0.864	0.885
MT-TMSE	0.930	0.810	0.823	0.887	0.884
WT-MT-CEEF	0.930	0.846	0.821	0.881	0.886
WT-MT-MSE	0.929	0.839	0.824	0.887	0.888
Article method	0.931	0.864	0.830	0.891	0.893

**Fig. 6:**
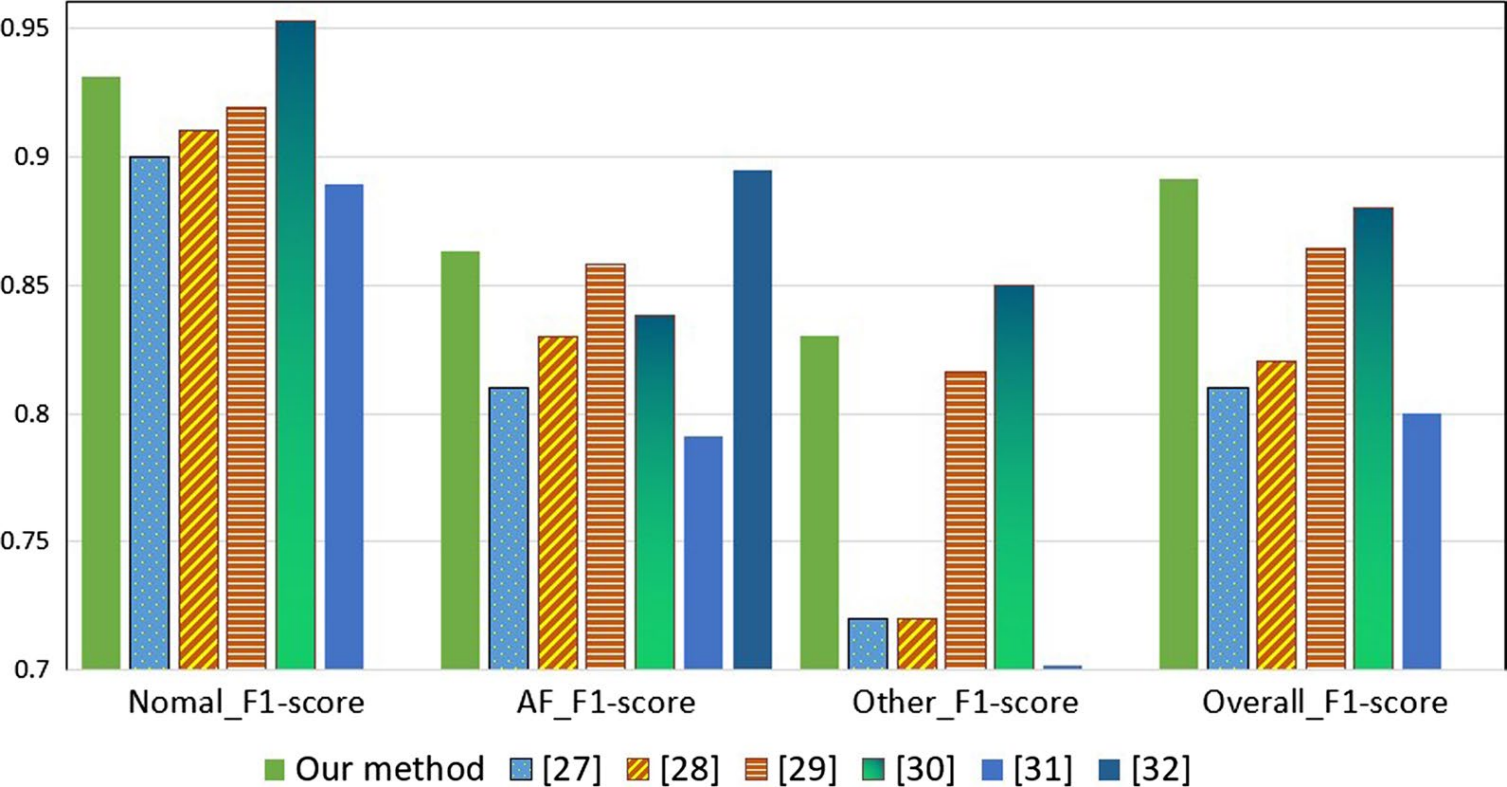
Method comparison char

8CSL [29] proposed a combination of a fast-connected 8-layer convolutional neural network (cnn) and a single-layer long short-term memory (LSTM) to classify ECG data. This method can extract features well. However, this method divides the ECG signal in the preprocessing stage, artificially reducing the timing characteristics of the ECG signal, and the loss decreases slowly, which is not conducive to training.Multi-SVM [22] introduced a new system that uses SVM classifier to classify ECG fluctuations. First, ECG is preprocessed. After ECG preprocessing, theQRS complexesare detected and segmented. Our decision rule uses dynamic reject thresholds following the cost of misclassifying a sample and the cost of rejecting a sample. But in the comparison of Other-F1-score, a lower score was achieved. Double-layer independent CNN [23] directly uses a simple convolutional neural network. The classification effect has improved significantly, especially on the ECG data of the AF category and the Other category.This method improves F1-score by more than 3$\%$ on the AF category data, and improves F1-score by 11$\%$ on the ECG data of the Other category. XGBoot and LSTMs stacked by LDA [25]takes raw ECG data (sampled at 200 Hz, or 200 samples per second) as input, and outputs a predicted value every 256 samples (or every 1.28 s), which is called the output interval. The network only takes raw ECG samples as input, and does not consider other patient or ECG-related features. The network architecture has 34 layers. In order to make the optimization of the network easier to process, a similar residual network architecture is used. The network is composed of 16 residual blocks, and each residual block spans two convolutional layers. The score of this method in Nomal-F1-score exceeds the method proposed in this article. Decision tree ensemble [26] involves extracting features from the ECG waveform and training a machine learning classifier.In feature extraction, standard feature selection methods are used to reduce the dimensionality of the feature space, and statistical features related to ECG signals and reference points are calculated. Sparse coding is used as an unsupervised feature extraction tool, and the classifier is a decision tree-a classifier based on ensemble learning.The histogram of experimental comparison is shown in Fig. 6, and the experimental data is shown in Table 5.

**Table 5:** Method classification results

Method	F1-score				Accurary
	Normal	AF	Other	Overall	
Multi-SVM [22]	0.90	0.81	0.72	0.81	
Double-layer independent CNN [23]	0.91	0.83	O.72	0.82	
21-layer 1D CNN [24]	0.919	0.858	0.816	0.864	
XGBoost and LSTMs stacked by LDA [25]	0.953	0.838	0.850	0.880	
Decision tree ensemble [26]	0.889	0.791	0.702	0.80	
8CSL [29]	–	0.895	–	–	
Article method	0.931	0.864	0.830	0.891	0.893

## Conclusion

In this paper, in the ECG data preprocessing, the combination of wavelet transform and median filtering is used. Among them, the wavelet transform uses the wavelet function to decompose the electrocardiogram signal into sub-signals of different frequency scales, and then performs the wavelet reconstruction after the segmentation filtering. For feature extraction, a DCNN and Bilstm are used for feature extraction. A 24-layer DCNN and cross-convolution kernels of different sizes are used for feature extraction. Dropout and batch normalization are used to transfer feature information Prevent data from overfitting. Combined with two layers of Bilstm to better fit the time-sensitive features of ECG data, and finally use softmax classifier for classification. In terms of the loss function, we propose a new loss function, which can better reflect the loss of model training to correct the optimization direction in time, thereby improving the classification accuracy of ECG data. This method has been verified on the ECG data set provided by the 2017 PhysioNet/CinC Challenge. Its accuracy is 0.893, and its F1 score is 0.891. Experiments show that the loss function we proposed can better fit the fluctuations in ECG training and cooperate with data denoising. Can effectively improve the accuracy of ECG recognition classification.

## Discussion

In this paper, the deep neural network is applied to the automatic classification of ECG, and different structures of deep neural network are used to extract the abstract features and location relevance of ECG signals. Through the experimental verification, good classification results have been achieved in the existing work, but there are still many shortcomings.

- According to the characteristics of ECG signal, the filtering algorithm used in this paper has a good filtering effect on the ECG data set provided by 2017 PhysioNet/CINC challenge, but the filtering effect will decline for the data collected by ECG acquisition instruments from different manufacturers. Next, we will study more robust preprocessing methods in data preprocessing.
- Due to the limitation of hardware equipment, the layers of bidirectional long-term and short-term memory network and convolutional neural network used in multi input feature fusion are relatively shallow. In addition, there are many excellent deep neural network models that can be used to experiment, and better network models can be used to realize the classification of different electrical categories.
- In the future, we hope to transplant the classification algorithm in this paper to the portable terminal device, realize the dynamic monitoring of ECG changes, provide reference for the prevention and treatment of heart disease, and make greater contribution to the diagnosis and treatment of cardiovascular disease.

## Data Availability

All data generated or analysed during this study are included in this published article [28].

## Abbreviations

AF: Atrial fibrillation
DCNN: Deep convolutional neural network
BiLSTM: Bi-directional long shortterm memory
TMSE: Tan mean square error
EMG: Electromyogram
ECG: Electrocardiogram
WT: Wavelet transform
MT: Median filter
CNN: Convolutional neural network
MSPCA: Multi-scale principal component analysis
DBN: Deep belief network
DAE: Denoising automatic encoder
